# Gender Differences and Socioeconomic Status in Relation to Overweight among Older Korean People

**DOI:** 10.1371/journal.pone.0097990

**Published:** 2014-05-16

**Authors:** Jin-Won Noh, Minkyung Jo, Taewook Huh, Jooyoung Cheon, Young Dae Kwon

**Affiliations:** 1 Department of Healthcare Management, Eulji University, Seongnam, Korea; 2 Graduate School of Public Health, Korea University, Seoul, Korea; 3 Strategy Department for Women-friendly Policy, Korean Women’s Development Institute, Seoul, Korea; 4 School of Nursing, University of Maryland, Baltimore, Maryland, United States of America; 5 Department of Humanities and Social Medicine, College of Medicine and Catholic Institute for Healthcare Management, the Catholic University of Korea, Seoul, Korea; CUNY, United States of America

## Abstract

**Background:**

The ever-increasing older population and its association with serious overweight problems have garnered much attention. The correlation between being overweight and socioeconomic status factors could be helpful for understanding the inequalities among the overweight population. We examined the correlation between being overweight and some key variables, such as demographics, socioeconomic status, general health status, and health behavior in a large sample of older individuals, by each gender.

**Methods:**

We used data from the 2008 Korean Longitudinal Study of Aging and it included 8,157 participants who were 45 years or older. To understand the relationship between the overweight participants in accordance to demographic and socioeconomic characteristics, health status, and health behaviors, a weighted chi-square test and logistic regression analysis were conducted by separating variables related to overweight, according to the genders.

**Results:**

The number of people in the normal group was 6,347 (77.8%), while the people who were considered overweight were 1,810 (22.2%). Women (n = 4,583) constituted 52.7% of the subject, 24.9% of whom were classified as overweight. Meanwhile, 20.6% of the 47.3% (n = 3,574) of the sample who were men were classified as overweight. Participants between the ages of 45 and 64 with chronic diseases were more likely to be overweight. Men in the 4th quartile of household income were more likely to be overweight than those who were in the 1st quartile, in contrast, while unemployed women with lower education levels and urban residents were at greater risk for being overweight.

**Conclusions:**

Among the men, health status and health behavior appeared to show a correlation with being overweight; however, among women, socioeconomic status factors were strongly related to being overweight. These findings appear to support the association of gender-specifics with the prevalence of being overweight.

## Introduction

The increasing trend of overweight individuals has become a major health issue over the past several decades [1]. According to the World Health Organization (WHO), it was estimated that more than 1.4 billion adults were overweight in 2008, and among them, at least 500 million were obese. This was an increase of over 200% since 1980 [1].

South Korea is one of the fastest industrialized countries. The prevalence of overweight among Koreans has increased during the past several decades due to socioeconomic development and the increase in life expectancy [2], [3]. According to the Korea National Health and Nutrition Examination Survey data, the prevalence of overweight [body mass index (BMI), 25.0–29.9 kg/m^2^] increased from 26.7% in 1998 to 30.9% in 2009.

Since Sobal and Stunkard's exhaustive review comparing the association between socioeconomic status (SES) and overweight [4], it was found that there may be a correlation between being overweight and economic development. There is an inverse association between overweight and SES among women in developed societies, whereas the association is direct in developing countries [5]–[7]. These findings stress significant gender differences in the association of overweight prevalence with SES factors [7].

SES factors, such as education level, income, and employment status, have a differential effect on body weight and fat distribution [8], [9]. Generally, people who were considered to have lower education were more highly associated with being overweight [10]–[12]. Some studies report that women with a lower education level are more likely to have a significantly higher BMI than compared to those with a higher education levels, even though there are no significant differences that were found in men [8], [13], [14]. Higher income was also found to have an association with a higher risk of being overweight [10]. Men with higher income typically have a greater BMI and a higher risk of being overweight than those who earn less income [8], [15], [16]. In contrast, for women, the prevalence of being overweight increased with lower income [15], [16]. Ball, Mishra, and Crawford reported that low-status employed women showed the higher risk of being overweight than high-status employed women [17]. However this finding was not observed among men. A study reported that SES, represented by income and education levels, was positively associated with overweight in Korean men, whereas a negative association between SES and overweight was found in Korean women [18]. A study of people in a rural community in Korea reported that the prevalence of overweight was higher among men with higher income as well as less education [19].

Interestingly, marital status is also an important factor that affects overweight. Married individuals are more likely to have higher BMI or have a higher risk of being overweight, particularly men [12], [13], [20], [21]. Also, location of residence was considered to be a factor because of the differential associated diet, physical activity, and environment (urban vs. rural) [14]. However, the relationship between location and being overweight was not evident. Padez reported that adult males who lived in rural areas had a lower risk of being overweight than those who lived in urban area [22], while a study by Tchicaya and Lorentz showed that adults who lived outside of urban areas were at a higher risk of being overweight [14].

Overall, gender differences in relation to the association between overweight prevalence and SES factors would be helpful for understanding the inequalities in being overweight; however, there is a lack of information about what actual risk factors affect the overweight occurrence among older people by gender in Korea [23]. Therefore, the aim of this study is to examine gender differences in the risk factors of being overweight, focusing on the SES factors among Koreans over 45 years of age. It is hypothesized that being overweight is associated with the SES, but the pattern of association would be different by gender.

## Methods

### 1. Data and Subjects

We used data from the 2008 Korean Longitudinal Study of Aging (KLoSA), which was obtained from a public repository (http://survey.keis.or.kr/survey_keis/). The Korea Labor Institute conducted KLoSA, funded by the Korean Ministry of Labor, to identify the aging progress of Koreans and to utilize its findings in academic research and policy-making. KLoSA has gathered data using computer assisted personal interviewing. The response rate is 86.6% and hot deck imputation method has been used to deal with missing data. The population of KLoSA participants includes adults aged 45 and over. The current study was carried out using data from 8,157 of the original 8,688 KLoSA subjects, after excluding subjects who were underweight or were missing BMI data. Since the database contains only de-identified data, this study does not pose any risks to study subjects. This study was also approved by the Institutional Review Board of the Catholic University of Korea with a waiver for informed consent because the data obtained from a public database.

### 2. Variables

#### Dependent variables

Based on WHO criteria, people with BMI of less than 18.5 kg/m^2^ (underweight) were excluded from the analysis because this research focused only on the overweight. People with a BMI from 18.5 to 25 kg/m^2^ were classified as ‘normal’ and those with a BMI of 25 kg/m^2^ or greater was considered as ‘overweight’. Other independent variables were defined using a questionnaire survey of the KLoSA.

#### Independent variables

Demographic variables included: age and gender. Socioeconomic variables included: marital status, level of education, residence, general quality of life (QOL), employment, household income, and medical insurance type. Marital status was classified into two groups: married and other status (divorced, parted by death, separated, single). Level of education was divided into four levels: college or higher than high school, middle school diploma, elementary school and less than elementary school. Relative QOL was assessed through peer comparison using a 10-point interval scale from 0 to 100 points. A higher score meant a higher QOL and the QOL range was divided into three categories at the less than 60, 60–80, and more than 80 point levels. Economic status was assessed based on annual household income in the previous year (2007). Medical insurance type was divided into National Health Insurance and Medical Aid.

Health status variables included: self-rated health, whether they have depressive symptom, whether they have disabilities, and whether they have chronic disease. The presence of depressive symptoms was assessed by the Korean version of the Center for Epidemiologic Studies Depression Scale (CES-D) survey. A simplified form of the CES-D, the CES-D10 is a questionnaire composed of 10 questions and was developed as a depressive symptom screening scale for epidemiological investigations. In the Korean version of CES-D10, answers to the question on the frequency of experiencing depressive symptoms during the past week were composed of four choices: ‘for a while’ (less than one day), ‘sometimes’ (from one to two days), ‘often’ (from three to four days), and ‘at all times’ (from five to seven days). We assigned answers of ‘for a while’ and ‘sometimes’ 0 points, and ‘often’ and ‘at all times’ 1 point, and set a cutoff of more than 4 points in 10 questions as having depressive symptoms. According to medical certification of their disability, the respondents were divided into two groups: impaired and non-impaired. We determined that a patient has chronic disease if he/she has at least one of the following items related to chronic illness: high blood pressure, diabetes, cancer, chronic lung disease, liver disease, heart disease, cerebrovascular disease, arthritis/rheumatism, prostate disease, and urinary incontinence.

Health behavior included: whether the respondents exercised, smoked, and/or drank. Respondents who answered yes to the question, “Do you usually exercise more than once a week?” were classified as the ‘regular exercise group,’ while those who said no to the same question were classified as the ‘no exercise group.’ Regarding smoking, the respondents were classified into current smokers, former smokers, and non-smokers. The same standard was applied to drinking.

### 3. Statistical Analysis

The chi square test and simple logistic regression analysis were conducted in order to determine the relationship among socio-demographic characteristics, health status and health behaviors based on gender and to identify the variables that show differences in normal weight and overweight by gender. Interaction effect analysis was examined between gender and the rest of the variables, and stratified analysis was conducted for each gender. In order to identify the factors that influence overweight based on gender, two models of multivariable logistic regression analysis was conducted for each gender group. Model 1 showed the association of age and socioeconomic position with overweight varied by gender. Model 2 included the addition of variables of health status and health behavior. All of the statistical analyses were two sided, and *P*<0.05 was considered to be significant. In order to generalize the analysis results through a sample survey, the KLoSA analyzed the survey results by subject, applying the weighted value which was calculated by taking into account the benchmarking adjustment values using extraction rate, response rate, and other external information. For all statistical analyses in this study, SAS ver. 9.2 (SAS Institute, Cary, NC, USA) was used, and all analyses were tested at a 95% confidence level.

## Results

### 1. General Characteristics of Subjects

Out of the total 8,157 subjects, the number of people in normal group was 6,347 (77.8%), while that of people who were overweight was 1,810 (22.2%). In the case of marital status, men were higher than women in percentage. Significantly more men (19.7%) than women (5.3%) had an education level of college or higher, and, naturally, fewer men (23.5%) than women (50.3%) had less than an elementary school education. With employment, men recorded a much higher employment rate of 69.2% than women’s 34.1%. Likewise, in self-rated health, 50.2% of men reported excellent or good health in contrast to 33.7% of women who reported the same. For such health status variables as depression, disability and chronic diseases, women were disproportionately associated with negative health factors, and men were more likely to be smokers and drinkers. In other areas such as residence, general QOL, household income, medical insurance, and exercise, men and women showed similar rates of distribution to each other (Table 1).

**Table 1:** Characteristics of the study population (n = 8,157), Korea, 2008*.

Variables	Subcategory	Both genders		Male	Female	*p*-value
		Before adjustment	After adjustment	After adjustment†	After adjustment‡	
		N (%)	%	%	%	
Body mass index (kg/m^2^)	18.5–25	6,347 (77.8)	77.1	79.4	75.1	<.001
	≥25	1,810 (22.2)	22.9	20.6	24.9	
Age (yrs, mean = 63.06)	45–54	2,208 (27.1)	38.0	40.4	35.9	<.001
	55–64	2,333 (28.6)	30.4	32.2	28.8	
	65–74	2,340 (28.7)	21.5	20.0	22.9	
	≥75	1,276 (15.6)	10.0	7.4	12.4	
Marital status	Married	6,410 (78.6)	81.7	91.5	72.8	<.001
	Widowed/divorced/single	1,747 (21.4)	18.3	8.5	27.2	
Level of education	College or higher	808 (9.9)	12.1	19.7	5.3	<.001
	High school	2,263 (27.8)	32.8	39.5	26.8	
	Middle school	1,363 (16.7)	17.5	17.3	17.7	
	Less than elementary school	3,720 (45.6)	37.6	23.5	50.3	
Residence	Rural	1,948 (23.9)	21.3	20.2	22.2	0.033
	Urban	6,209 (76.1)	78.7	79.8	77.8	
General quality of life	<60	3,037 (37.2)	36.1	32.6	39.3	<.001
	60–79	3,158 (38.7)	39.4	41.0	37.9	
	≥80	1,962 (24.1)	24.5	26.4	22.8	
Employment	Yes	3,514 (43.1)	50.7	69.2	34.1	<.001
	No	4,643 (56.9)	49.3	30.8	65.9	
Household income	1st quartile	2,009 (24.6)	23.7	21.6	25.5	0.002
	2nd quartile	1,900 (23.3)	21.7	21.5	21.9	
	3rd quartile	2,203 (27.0)	26.7	27.6	25.9	
	4th quartile	2,045 (25.1)	28.0	29.2	26.8	
Medical insurance	Medical Aid	459 (5.6)	5.1	4.6	5.6	0.042
	National Health Insurance	7,698 (94.4)	94.9	95.4	94.4	
Self-rated health	Excellent	252 (3.1)	4.0	4.9	3.2	<.001
	Good	2,667 (32.7)	37.5	45.3	30.5	
	Fair	2,980 (36.5)	34.8	32.8	36.6	
	Poor	1,892 (23.2)	19.9	14.4	24.9	
	Bad	366 (4.5)	3.8	2.7	4.7	
Depression	Yes	3,782 (46.7)	42.4	35.9	48.2	<.001
	No	4,325 (53.4)	57.6	64.1	51.8	
Disability	Yes	2,623 (32.2)	28.0	21.7	33.7	<.001
	No	5,534 (67.8)	72.0	78.3	66.3	
Chronic diseases	Yes	3,648 (44.7)	40.1	35.4	44.3	<.001
	No	4,509 (55.3)	59.9	64.6	55.7	
Exercise	Yes	2,978 (36.5)	37.6	40.0	35.4	<.001
	No	5,179 (63.5)	62.4	60.0	64.6	
Smoking	Smoker	1,476 (18.1)	21.2	41.6	2.9	<.001
	Ex-smoker	993 (12.2)	12.0	24.4	1.0	
	Non-smoker	5,687 (69.7)	66.7	34.0	96.2	
Drinking	Drinker	3,029 (37.1)	42.3	66.0	21.0	<.001
	Ex-drinker	777 (9.5)	8.9	13.7	4.6	
	Non-drinker	4,351 (53.3)	48.8	20.3	74.4	

*The KLoSA as a sample survey was analyzed by research subject and with applied weight calculated in consideration of benchmarking adjustment values that use extraction rate, response rate, and other external information. †Sample size = 3,574 weighted = 6,931,093. ‡Sample size = 4,583 weighted = 7,709,410.

### 2. Relationships between Demographic and Social Characteristics and Health Behaviors with Overweight

Table 2 displays the results from chi square test which indicates which variables among socio-demographic characteristics, health status, and health behaviors show differences in normal weight and overweight by gender. Results from examining the interaction effect between gender and the rest of the variables showed that most of the variables had significant interaction effect except for marital status, residence, and drinking. Residence, employment, and chronic diseases were found to have a significant relationship with being overweight in both men and women. As for age, QOL, household income, insurance, depression (CES-D10), and exercise, a significant relationship was identified in men, but not women. Level of education, self-rated health, and presence of a disability showed significance only among women (*P*<0.05) (Table 2).

**Table 2:** Chi square test on demographics, socioeconomic characteristics, and health behaviors with overweight.

Variables	Subcategory	Male†			Female‡			Interaction *p*-value
		18.5≤ BMI <25 (%)	25≤ BMI (%)	*p*-value	18.5≤ BMI <25 (%)	25≤ BMI (%)	*p*-value	
Age (yrs)	45–54	39.5	43.8	<.001	37.6	30.7	<.001	
	55–64	31.6	34.4		27.5	32.8		0.010
	65–74	20.8	17.1		21.7	26.3		<.0001
	≥75	8.1	4.7		13.1	10.1		0.002
Marital status	Married	91.0	93.4	0.056	73.1	71.8	0.429	
	Widowed/divorced/single	9.0	6.6		26.9	28.2		0.128
Level of education	College or higher	19.8	19.4	0.290	6.0	3.0	<.001	
	High school	38.6	42.8		28.1	22.8		0.211
	Middle school	17.5	16.7		17.2	19.4		0.007
	Less than elementary school	24.1	21.2		48.7	54.9		0.001
Residence	Rural	20.9	17.4	0.045	23.3	19.0	0.003	
	Urban	79.1	82.6		76.7	81.1		0.859
General quality of life	<60	33.6	28.6	0.031	38.9	40.6	0.638	
	60–79	41.0	41.3		38.1	37.1		0.078
	≥80	25.4	30.1		23.0	22.3		0.005
Employment	Yes	68.0	73.7	0.005	35.6	29.6	0.001	
	No	32.0	26.3		64.4	70.4		<.0001
Household income	1st quartile	22.7	17.5	<.001	24.8	27.5	0.332	
	2nd quartile	22.1	19.6		21.8	22.0		0.215
	3rd quartile	27.9	26.3		26.1	25.3		0.065
	4th quartile	27.3	36.7		27.3	25.3		<.0001
Medical insurance	Medical Aid	5.0	2.8	0.015	5.3	6.6	0.112	
	National Health Insurance	95.0	97.3		94.7	93.4		0.004
Self-rated health	Excellent	4.7	5.6	0.277	3.6	2.2	<.001	
	Good	44.4	48.5		32.7	24.2		0.547
	Fair	33.6	30.0		36.2	37.9		0.028
	Poor	14.7	13.2		22.9	30.9		0.004
	Bad	2.7	2.6		4.7	4.9		0.122
Depression	Yes	37.4	30.4	0.002	47.9	49.1	0.518	
	No	62.6	69.6		52.1	50.9		0.006
Disability	Yes	22.3	19.3	0.103	31.5	40.1	<.001	
	No	77.7	80.7		68.5	59.9		<.0001
Chronic diseases	Yes	33.3	43.8	<.001	39.3	59.4	<.001	
	No	66.8	56.2		60.7	40.6		0.001
Exercise	Yes	38.3	46.5	<.001	34.9	36.8	0.312	
	No	61.7	53.5		65.1	63.2		0.038
Smoking	Smoker	42.7	37.5	0.066	2.7	3.4	0.504	
	Ex-smoker	24.2	25.2		1.0	1.0		0.444
	Non-smoker	33.1	37.4		96.4	95.7		0.044
Drinking	Drinker	65.8	66.7	0.651	20.7	22.0	0.344	
	Ex-drinker	13.5	14.3		4.4	5.3		0.756
	Non-drinker	20.6	19.0		74.9	72.7		0.867

† Sample size = 3,574 weighted = 6,931,093.

‡ Sample size = 4,583 weighted = 7,709,410.

### 3. Factors Affecting Overweight among Demographic and Social Characteristics and Health Behaviors

In the case of men, the overweight OR did not significantly decrease for those in the 55–64 age group compared to the 45–54 age group, but did for the 65–74 age group and the over 75 age group. Thus, the OR of overweight decreased with older age. The findings showed that the overweight OR was higher for those in the 4th quartile of household income than the 1st quartile in Model 1 and Model 2. The result of Model 2 for men showed that age, level of education, household income, chronic diseases, and exercise were significant variables. It was found that men with less education than elementary school level were more likely to belong to the overweight group compared to men from college or higher (OR, 1.51; 95% CI, 1.08–2.13). The probability of being overweight among men increased significantly with chronic diseases (OR, 1.94; 95% CI, 1.56–2.42) and exercise (OR, 1.06; 95% CI, 1.01–1.12) (Table 3).

**Table 3:** Multivariable logistic regression analysis on BMI [for 25 kg/m^2^≤ BMI (compared to 18.5 kg/m^2^≤ BMI <25 kg/m^2^)].

Variables	Subcategory	Male								Female							
		Model 1				Model 2				Model 1				Model 2			
		OR		95% CI		OR		95% CI		OR		95% CI		OR		95% CI	
Age (yrs)	45–54	1 (Reference)				1 (Reference)				1 (Reference)				1 (Reference)			
	55–64	0.94	0.74		1.19	0.87	0.68		1.11	1.23	0.99		1.53	1.04	0.83		1.30
	65–74	0.74	0.56		0.98	0.65	0.49		0.87	1.13	0.89		1.44	0.83	0.64		1.07
	≥75	0.56	0.38		0.83	0.49	0.33		0.74	0.68	0.50		0.92	0.50	0.36		0.69
Marital status	Widowed/divorced/single	1 (Reference)				1 (Reference)				1 (Reference)				1 (Reference)			
	Married	1.10	0.77		1.59	1.03	0.71		1.50	1.00	0.83		1.20	0.99	0.82		1.20
Level of education	College or higher	1(Reference)				1(Reference)				1(Reference)				1(Reference)			
	High school	1.28	0.97		1.70	1.34	1.00		1.78	1.71	1.09		2.67	1.66	1.06		2.60
	Middle school	1.23	0.87		1.74	1.34	0.94		1.91	2.33	1.48		3.69	2.13	1.34		3.38
	Less than elementary school	1.39	1.00		1.93	1.51	1.08		2.13	2.51	1.59		3.97	2.11	1.33		3.35
Residence	Rural	1 (Reference)				1 (Reference)				1 (Reference)				1 (Reference)			
	Urban	1.17	0.92		1.47	1.10	0.87		1.40	1.42	1.17		1.71	1.36	1.12		1.65
General quality of life	<60	1(Reference)				1(Reference)				1(Reference)				1(Reference)			
	60–79	1.03	0.80		1.33	1.02	0.79		1.32	1.02	0.86		1.22	1.05	0.88		1.27
	≥80	1.18	0.90		1.56	1.12	0.84		1.50	1.05	0.86		1.30	1.08	0.87		1.34
Employment	Yes	1 (Reference)				1 (Reference)				1 (Reference)				1 (Reference)			
	No	0.95	0.75		1.19	0.85	0.67		1.08	1.26	1.05		1.51	1.19	0.98		1.44
Household Income	1st quartile	1 (Reference)				1 (Reference)				1 (Reference)				1 (Reference)			
	2nd quartile	1.06	0.78		1.44	1.00	0.73		1.37	0.90	0.73		1.13	0.92	0.73		1.15
	3rd quartile	1.10	0.81		1.49	1.05	0.77		1.43	0.88	0.70		1.10	0.92	0.73		1.15
	4th quartile	1.60	1.16		2.21	1.46	1.05		2.03	0.89	0.70		1.14	0.93	0.72		1.19
Medical insurance	Medical Aid	1 (Reference)				1 (Reference)				1 (Reference)				1 (Reference)			
	National Health Insurance	1.09	0.96		1.25	1.09	0.95		1.25	0.97	0.90		1.05	0.98	0.90		1.07
Self-rated health	Excellent					1 (Reference)								1 (Reference)			
	Good					1.02	0.63		1.64					1.18	0.66		2.10
	Fair					0.89	0.54		1.46					1.45	0.82		2.58
	Poor					0.97	0.55		1.70					1.44	0.80		2.59
	Bad					1.24	0.57		2.68					1.01	0.52		1.98
Depression (CES-D10)	Yes					1 (Reference)								1 (Reference)			
	No					1.24	0.99		1.55					1.17	0.99		1.38
Disability	No					1 (Reference)								1 (Reference)			
	Yes					0.91	0.67		1.22					1.25	1.02		1.52
Chronic diseases	No					1 (Reference)								1 (Reference)			
	Yes					1.94	1.56		2.42					2.16	1.82		2.56
Exercise	No					1 (Reference)								1 (Reference)			
	Yes					1.06	1.01		1.12					1.02	0.97		1.06

OR, odds ratio; CI, confidence interval; QOL, quality of life.

On the other hand, when compared to the 45–54 age group women, the OR of overweight was significantly lower only in the over 75 age group. Women with high school, middle school, and less than elementary school level education were more likely to be overweight than those who had a college or higher degree and the proportion of overweight was higher among women with living urban in both Model 1 and Model 2. Unemployed women showed a higher risk of being overweight (OR, 1.26; 95% CI, 1.05–1.51) in Model 1. Age, level of education, residence, disability, and chronic diseases were significant variables among women in Model 2. The proportion of being overweight was higher women with a disability (OR, 1.25; 95% CI, 1.02–1.52) and women with chronic diseases (OR, 2.16; 95% CI, 1.82–2.56) (Table 3).

## Discussion

In this study we examined the correlation between overweight and some key variables, such as demographics, SES, health status, and health behaviors, in a large sample of older individuals (over 45 years of age). This research showed first that health status (chronic diseases) and age had a higher correlation with the risk of being overweight – particularly, younger men and women. Among men, those who exercise were more likely to be overweight, whereas among women, SES factors were strongly related to being overweight. In addition, among men, household income was strongly associated with being overweight. Among women, the level of education (high school, middle school, less than elementary school) was significantly related to being overweight in both Models 1 and 2. In short, these findings may support gender-specific associations with the prevalence of overweight.

Regarding the demographics, we found that age may have a relationship with the increased risk of being overweight – being in inverse proportion. While the prevalence of being overweight in the 65 years and older decreased relatively, the prevalence of overweight in the 45–64 age group increased. This result was not consistent with previous research that supports the association that the prevalence of overweight increases with age – directly proportional [10], [11], [13], [24]–[26]. One possible explanation for our results is that environmental factors, such as cultural background and dietary habits, may be contributing factors [27]. For example, Koreans in the 45–64 age group may appear to more frequently engage in lifestyles involving heavy dinners and alcohol consumption. In addition, office workers in Korea usually drink to increase sociability and reduce tension [28]. According to a survey conducted in 2005, 53.3% of white-collar employees in Korea go for a drink with colleagues more than twice a week, and 36.2% of employees have an incidence of heavy drinking once in a week [29].

In relation to SES factors, the following important findings were revealed. The prevalence of being overweight appears to be significantly associated with a lower level of education than college or higher, particularly among women. We found that women with a lower level of education had higher significant relationship with overweight than their more educated counterparts (‘high school diploma’). This is consistent with the results of a large number of studies that demonstrate that being female with a high SES in a developed country is inversely related to being overweight [17], [18]. This result is in line with the idea that females with upper SES (including the employed with higher level of education) may be more interested in maintaining a good body figure for achieving greater confidence and work efficiency as well. In addition, it may reflect another fact that women in Korea used to be discriminated against in terms of education. In the past, the disparities in education background between men and women were huge, and only a few women could attain a high level of education. Therefore, levels of education and social activities among women (rather than men) have a more positive association with a physical and psychological well-being lifestyle. This also demonstrates that the Korean society places importance on physical appearances of women.

Also, we found that people living in urban areas were more likely to be obese, and females living in urban areas had an even stronger relationship with an increased risk of being overweight. As argued in a previous study [30], this relationship may be because urban dwellers tend to do less leisure-time physical activity and have less desirable eating habits. This relationship also shows that characteristics of the social environment may relate to trends in weight gain [12]. However, among men, there was no relationship with living in urban areas when we did multiple logistic regression analysis. It could be explained that the associations with other variables was stronger than that of the location of their home.

Meanwhile, in relation to the health status, although generally assumed, we particularly identified again that the critical factor associated with the risk of overweight, regardless of gender, appears to be chronic diseases. The presence of chronic diseases showed that those who have chronic diseases, regardless of age, are more obese than those without. Many studies have shown that overweight is strongly associated with an increased risk for many chronic diseases, including osteoarthritis, diabetes, hypertension, and hypercholesterolemia [27], [31].

However, there are several limitations in this study. Although household income only had no for female in this study, the association between level of income and the prevalence of overweight has been identified in previous research [8], [32]. In the current study we did not include income from property, which can be an important household income source. With more exact sources of household income (economic determinant) and social status factors that include various social activities and involvement in social and health groups, the current study could have even better defined the relationship between SES and the risks of overweight. Another limitation is that this study does not show time-series analysis since it is a cross sectional study. Therefore, through this research, any time series changes and findings are not analyzed and/or explained. Therefore, a longitudinal study that could further determine cause-outcome relationships is necessary. It can also be argued that the limitations of sampling survey are overcome from applying the weighted value to solve these problems of the sampling survey. Also, in terms of generalization of the above findings, it is critically required to look at different contexts of SES factors in different countries and societies as well. These limitations are areas for meaningful further study and the need for careful interpretation of the findings in this study.

## Conclusion

This study looked at the gender difference in the risk of being overweight in relation to SES among older Koreans. It reveals that there are some important gender-specific associations with the risk of overweight. While males were found to have a significantly more associations with health status and health behavior, such as chronic diseases and exercise, female were found to be strongly related to socioeconomic factors, such as education, residence, and employment status. Culturally, it is possible that female with upper SES may be more strongly required to manage her body shape than male. This study shows that socioeconomic factors contributing to being overweight are different by gender. Therefore, it is necessary to consider the different risk of SES by gender in implementing a comprehensive national policy or initiative program to reduce and prevent the increasing problem of being overweight.
